# Repurposing Quinacrine for Treatment of Malignant Mesothelioma: In-Vitro Therapeutic and Mechanistic Evaluation

**DOI:** 10.3390/ijms21176306

**Published:** 2020-08-31

**Authors:** Nishant S. Kulkarni, Bhuvaneshwar Vaidya, Vineela Parvathaneni, Debarati Bhanja, Vivek Gupta

**Affiliations:** 1 Department of Pharmaceutical Sciences, College of Pharmacy and Health Sciences. St. John’s University, Queens, NY 11439, USA; nishant.kulkarni16@my.stjohns.edu (N.S.K.); vineela.parvathaneni16@my.stjohns.edu (V.P.); 2 School of Pharmacy, Keck Graduate Institute, Claremont, CA 91711, USA; bhuvan.vaidya@ttuhsc.edu (B.V.); dbhanja@pennstatehealth.psu.edu (D.B.); 3 School of Pharmacy, Texas Tech University Health Sciences Center, Amarillo, TX 79106, USA; 4 College of Medicine, Penn State University, Hershey, PA 15213, USA

**Keywords:** drug repurposing, quinacrine, malignant pleural mesothelioma, autophagy, apoptosis

## Abstract

Malignant mesothelioma (MM) is a rare type of cancer primarily affecting mesothelial cells lining the pleural cavity. In this study, we propose to repurpose quinacrine (QA), a widely approved anti-malarial drug, for Malignant Pleural Mesothelioma (MPM) treatment. QA demonstrates high degree of cytotoxicity against both immortalized and primary patient-derived cell lines with sub-micromolar 50% inhibitory concentration (IC_50_) values ranging from 1.2 µM (H2452) to 5.03 µM (H28). Further, QA also inhibited cellular migration and colony formation in MPM cells, demonstrated using scratch and clonogenic assays, respectively. A 3D-spheroid cell culture experiment was performed to mimic in-vivo tumor conditions, and QA was reported to be highly effective in this simulated cellular model. Anti-angiogenic properties were also discovered for QA. Autophagy inhibition assay was performed, and results revealed that QA successfully inhibited autophagy process in MPM cells, which has been cited to be one of the survival pathways for MPM. Annexin V real-time apoptosis study revealed significant apoptotic induction in MPM cells following QA treatment. Western blots confirmed inhibition of autophagy and induction of apoptosis. These studies highlight anti-mesothelioma efficacy of QA at low doses, which can be instrumental in developing it as a stand-alone treatment strategy for MPM.

## 1. Introduction

Malignant mesothelioma (MM) is a type of cancer that affects the mesothelium tissue lining found around major bodily organs like lungs, liver, heart, etc. [1,2] MM can be classified based on its type and location in the body. For instance, Malignant Pleural Mesothelioma (MPM) is the cancer that affects the pleural lining of the lungs, peritoneal mesothelioma affects the abdominal linings, whereas pericardial mesothelioma affects the lining of the heart [3,4,5]. MM is classified as a rare disease with about 3000 new cases diagnosed in the United States annually. Even though all bodily tissues are equally at risk, majority of the diagnosed cases reveal that lungs and chest wall are the most susceptible to MM. MM occurring in the chest wall is often referred to as pleural mesothelioma (MPM; pleura is a thin layer of tissue lining the lungs) and is predominantly seen to occur in geriatric patients with an average age of occurrence being 72 years [6]. Pleural mesothelioma accounts for 80–90% of all mesothelioma diagnosis. Occupational and accidental inhalation of asbestos fibers has been cited as one of the major cause for MPM onset in patients, leading to patient discomfort, difficulty in breathing, chest pain, etc. [7,8,9]. Initial stages of MPM are often associated with difficulty in breathing, which mainly is a result of pleural effusions (excess fluid buildup in the pleural area in the lungs); as the disease progresses, tumor in the affected pleural lining obstructs normal respiratory functioning leading to congestion and further breathing complications.

From a therapeutic perspective, surgical removal (resection) of tumor has not been very successful as it is nearly impossible to completely resect tumor from patients. Moreover, surgical procedures are today more focused on providing symptomatic relief via procedures like debulking pleurectomy, than intentions to cure MPM completely [10]. Having said that, a chemotherapy is more of a go-to approach when it comes to cancer therapeutics. MPM has been countered by use of a combination therapy involving platinum-based drugs (cisplatin) and antifolate drugs (pemetrexed (Alimta^®^)). This combinatorial therapy of cisplatin and pemetrexed was first tested in clinic in 2003 and it was successful in increasing patient survival from ~9 months to ~12 months [11]. Since then, there has not been any significant breakthrough in MPM chemotherapy, although recently in 2019, US Food and Drug Administration (USFDA) approved a novel electric-current based device therapy for MPM treatment, 15 years after cisplatin + pemetrexed combination. This therapy, developed by Novocure, called NovoTTF-100L™, uses low intensity electric fields, called Tumor Treatment Fields (TTF) to disrupt cellular divisions that occur rapidly in cancers [12]. The non-invasive therapy is however to be used with the standard cisplatin + pemetrexed chemotherapy, and the usage is only limited to patients with unresectable, locally advanced and metastatic MPM tumor [13]. The difficulty in achieving any significant breakthrough in chemotherapy can be attributed to the aggressive nature of MPM progression in lungs’ pleural lining, delayed diagnosis of asbestos inhalation and poor translatability of innovative therapies from bench to bedside. There is a growing need for innovative therapies to eradicate MPM from the society. In the USA alone, an estimated number of deaths rise as high as 3000/year with the number of occurrences increasing every year [14]. MPM globally affects ~40,000 patients/year [15] and the death toll is estimated to peak in the year 2020 [16].

As is evident, patient survival rate improved by 3 months for cisplatin + pemetrexed treated patients. While this is a great achievement, patient survival still needs to be drastically improved and ways to cure MPM completely need to be innovated. This may be made possible by exploring newer therapeutic approaches to discover new molecules with better therapeutic efficacy against MPM than current treatment regimens. One upcoming strategy for discovering new molecules is drug repurposing, an atypical strategy for discovering new drugs [17], which can be beneficial in bettering the MPM prognosis. This approach results in molecular reconfiguration of FDA-approved drugs, and assessing their efficacy against different disease conditions than their approved indication [18]. This strategy can end up saving millions of dollars in Phase-I clinical trials (safety testing) and can also help save up on a lot of time and efforts from translational perspective [19]. However, some dose-finding requirements may cite the need for Phase-I investigations. Keeping these benefits in mind, this study aims at repositioning a traditional anti-malarial drug, quinacrine (QA), for bettering current MPM therapy. Reports suggest multiple molecular targets for QA in different cancer types [20,21], which overlap in MPM and can potentially be exploited for therapeutic purposes. QA actively affects the apoptosis process in cancerous cells via activation of apoptotic proteins, like caspase-3/8/9 [22]. QA has also been reported to downregulate NF-kß and its associated pathways, leading to tumor growth inhibition. Apart from this, one unique pathway that QA affects is autophagy [22,23]. Autophagy is a mechanism by which cells recycle their cellular products and byproducts to generate energy in the form of ATP. Autophagy process kicks in during stressful cellular conditions, like hypoxia, enhanced metabolism during cancer, etc. [24]. Not only therapeutic efficacy but clinical safety also has been established for QA. Being an FDA approved anti-malarial drug, it has been extensively tested for its pharmacokinetic and safety profile, which cuts down the need for Phase-I clinical trials if repurposed for MPM treatment.

Drug repositioning often results in discovery of newer therapeutic approaches on a molecular level and this strategy has found excellent applications in discovery of novel MPM therapeutics [25,26]. There are multiple pathways involved in aggressive MPM progression, and it would be very interesting to evaluate the efficacy and molecular mechanism of QA activity in MPM. QA has been reported in several published reports to be effective against multiple types of cancers including non-small cell lung cancer, breast cancer, colorectal cancer, etc. [20,27,28]. MPM shares many common pathogenic pathways with these cancers as a part of its survival and progression mechanisms. These pathways include, but are not limited to, angiogenesis, autophagy, and evasion of apoptosis [29,30,31]. Several reports cite these three pathways as major pathways for MPM survival and progression. Based on these commonalities, in this study, a complete in-vitro efficacy and molecular marker profile of QA was assessed, and its potential to be utilized as a standalone therapy for MPM was evaluated.

## 2. Results and Discussion

### 2.1. Evaluation of Therapeutic Efficacy of Quinacrine (QA) in Treatment of Malignant Mesothelioma (MPM)

#### 2.1.1. QA Demonstrates Enhanced Cytotoxic Ability as Compared to CS in Immortalized and Patient-Derived MPM Cells

Approved in 2004, a combination of cisplatin + pemetrexed (Alimta^®^) is the only FDA-approved first-line chemotherapy for MPM treatment. In 2003, Vogelzang et al. demonstrated that this combination therapy improved the median patient overall survival from 9.3 months to 12.1 months; and, since then, this combination, approved by the FDA in 2004, has been adopted globally for improving MPM patient outcomes [11]. However, as is evident from <3-month improvement in overall survival, there is still room for further improvement, requiring novel therapeutic interventions.

**Figure 1:**
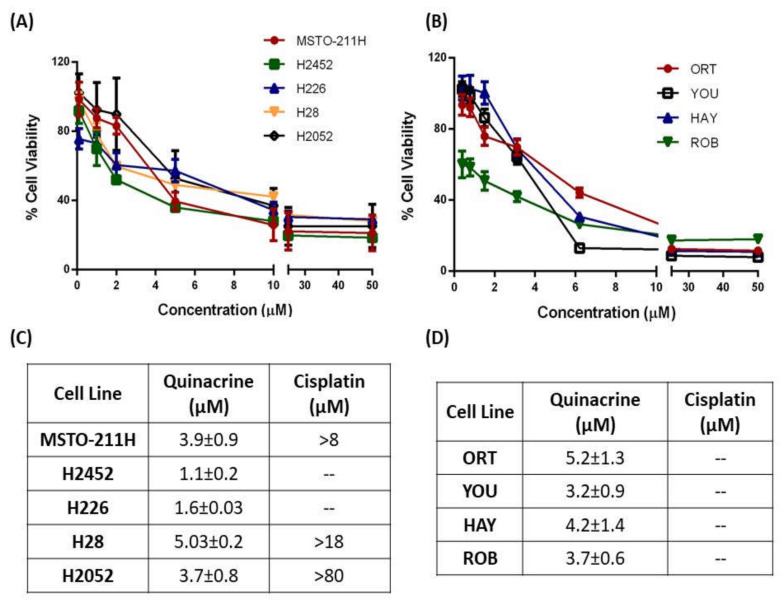
Cytotoxicity screen for quinacrine (QA) and cisplatin (CS) on immortalized and patient-derived primary MPM cells. (**A**) Concentration dependent cytotoxic activity of quinacrine (QA) in immortalized mesothelioma cell lines MSTO-211H, H2452, H226, H28, and H2052 and (**B**) patient-derived mesothelioma cell lines ORT, YOU, HAY, and ROB. Briefly, 2500 cells/well were plated in 96-well plates, and following overnight attachment, were exposed to various concentrations of either cisplatin or quinacrine for 72 h. After 72 h, cytotoxicity was measured with Cell Titer Blue assay kit. (**C**) Mean IC_50_ values for QA and cisplatin calculated from the cell toxicity data obtained using GraphPad Prism for all immortalized cell lines. (**D**) IC_50_ values for QA and cisplatin for all patient-derived cell lines Data are presented as % cell viability and represent mean ± SD (average of 3 individual experiments with *n* = 6).

Extensive cytotoxicity studies revealed concentration-dependent cytotoxicity and sub-micromolar IC_50_ values of QA in all mesothelioma cell lines, both primary and immortalized, indicating excellent efficacy (Figure 1). QA was compared with cisplatin (CA), a common drug used in platinum-based therapy of MPM. QA showed excellent efficacy at low does in immortalized cells like MSTO-211H (IC_50_: 3.9 ± 0.9 µM), H2452 (IC_50_: 1.1 ± 0.2 µM), H226 (IC_50_: 1.6 ± 0.03 µM), H28 (IC_50_: 5.03 ± 0.2 µM), and H2052 (IC_50_: 3.7 ± 0.8 µM) (Figure 1A,C). Cisplatin on the other hand showed minimal efficacy against all cell lines, with high IC_50_ values (e.g., H2052, IC_50_ 81.7 µM) (Figure 1C). In many cell lines, IC_50_ value for cisplatin could not be determined showing the degree of resistance to CS therapy. Building on these results, primary patient-derived cell lines were procured from Dr. Raffit Hassan’s laboratory at the National Institutes of Health (NIH). These primary cell lines were isolated from neoplastic effusions of patients undergoing therapeutic paracenteses; originally provided by The Stehlin Foundation (Houston, TX, USA) to the NIH; and were established as ORT, ROB, YOU, and HAY in a study by Li et al. [32]. Patient derived primary cell lines were used to establish a more physiologically relevant efficacy profile for both CS and QA, so as to have a better understanding of mesothelial cell behavior against QA therapy (Figure 1B). As can be seen from Figure 1B,D, QA showed similar sub-micromolar IC_50_ values in all patient-derived cell lines, ORT (IC_50_: 5.2 ± 1.3 µM), YOU (IC_50_: 3.2 ± 0.9 µM), HAY (IC_50_: 4.2 ± 1.4 µM), and ROB (IC_50_: 3.7 ± 0.6 µM). These results show immense potential of QA being developed as a standalone therapy for MPM treatment.

Effects of QA on normal cells’ viability were assessed by incubating different QA concentrations with normal human embryonic kidney cells (HEK-293). It can be seen from the data presented in Figure S1, QA was less or similarly toxic to normal cells as compared to cancer cells being studied in this project, that would highlight a potential need for developing a localized delivery system for the same, so as to limit exposure to normal tissues (Figure S1).

#### 2.1.2. QA Attenuates Colony Formation in MPM Cells: Clonogenic Assay

**Figure 2:**
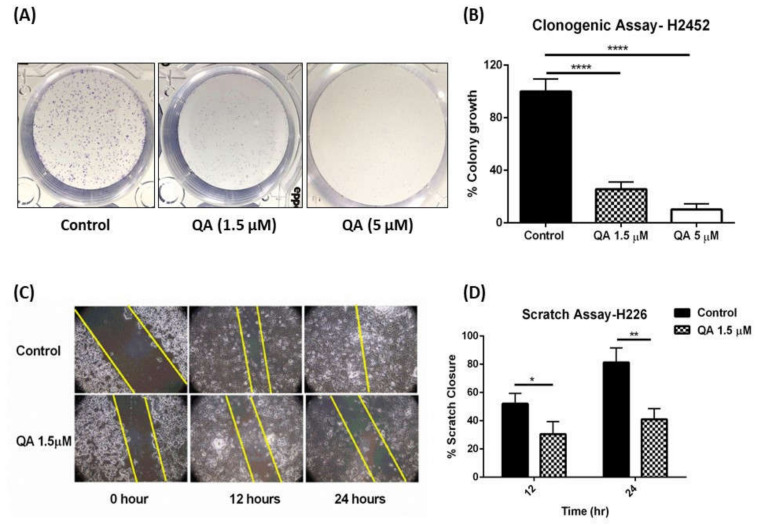
Evaluation of QA efficacy on colony formation and cellular migration in Malignant Pleural Mesothelioma (MPM) cells. (**A**) Clonogenic assay performed on H2452 cells with 500 cells/well with two concentrations of QA (1.5- and 5-µM). A concentration dependent inhibition of colony formation can be seen as 5 µM QA shows negligible formation of H2452 colonies. (**B**) Quantification of % colony growth per treatment relative to control. Significant difference between colony growth is seen between control group of cells and QA-treated cells. (**C**) Representation of scratch assay done on a confluent layer of H226 cells. Scratch is represented as yellow lines draw across the image. Scratch closure is more predominant in untreated cells in comparison to QA-treated cells. (**D**) Percent of scratch closure for control group of cells and QA (1.5 µM)-treated cells. A significant difference can be seen in % scratch closure at both time points between QA-treated and untreated cells. Data represent mean ± SD (*n* = 3), * *p* < 0.05, ** *p* < 0.01, **** *p* < 0.0001 compared between treatments and control groups, as indicated.

Surgical resection, also known as macroscopic complete resection (MCR) of tumorous mass associated with malignant mesothelioma, is one of the most common go-to interventions for its treatment; with pre-, intra-, and postoperative chemotherapy (multimodal therapy) [33]. However, surgical removal is often associated with high chances of condition relapse with as high as 77–80% chances of tumor recurrence [34,35]. The main reason for this relapse, usually local in origin, could be attributed to inability to completely eradicate cancerous cells during MCR, leaving behind small remnants of cancer cells [35,36,37]. These cells have the ability to either grow locally, or to metastasize by initially forming colonies and establishing contact with other cancerous cells. The effect of treatment on inhibition of this colony formation can be assessed by performing a simple clonogenic assay [38]. Clonogenic assay helps in determining the extent of inhibition of colony formation and gives insight into the probable post-operative behavior of cells. This study not only establishes the efficacy of QA in MPM but also hints at its potential use as a post-operative treatment for maintaining tumor free survival. To evaluate a post-operative scenario in-vitro, clonogenic assay was performed which involved plating small number of cells in culture plates and allowing high incubation time and colony formation. In this assay, QA (1.5- and 5-µM) was tested for its anti-colony formation ability in H2452 cells, known to have the ability to form colonies [39]. As can be visually seen from representative images shown in Figure 2A, there was a concentration dependent inhibition of H2452 colonies after a 48-h treatment with QA. Upon colony counting and normalizing the data relative to no treatment control (100% colony formation), 1.5 µM QA demonstrated only 25.6 ± 5.5% colony growth, and 5 µM had a mere 10.2 ± 4.4% colony growth as compared to control (*p* < 0.0001) (Figure 2B). This shows that QA is effective in inhibiting colony formation outlining its potential efficacy as a post-resection maintenance therapy for MPM.

#### 2.1.3. QA Therapy Results in Inhibition of Cellular Migration in MPM Cells: Scratch Assay

Cellular migration is another way by which the cancer cells establish contact with other tumorous cells, leading to metastasis in patients. If not controlled, migration of cancerous cells can have detrimental effects, as it is highly prevalent in post-operative conditions and can lead to tumor recurrence [40]. To assess the efficacy of a therapy toward inhibition of cellular migration, scratch or a wound healing assays are usually performed. Manually, a scratch is drawn across a confluent layer of cells and cancerous cells are then allowed to migrate to fill up the scratch. Ability of treatment groups to inhibit the scratch closure is evaluated by microscopic evaluation. Though not completely, this assay simulates physiological conditions of cellular migration in-vitro very closely [41]. To test the inhibitory effect of QA on cellular migration, scratch assay was performed using confluent H226 cells. Scratch was drawn across the well as described in the Methods section, followed by treatment with QA (1.5 µM) and the wells were imaged at 0, 12, and 24 h. It can be seen, in Figure 2C, that QA treatment inhibited cellular migration of H226 cells, outlined by rather consistent breadth of the scratch, as compared to control (no treatment) cells where scratch almost completely closed at 24 h (Figure 2C). Image quantification revealed that QA significantly inhibited scratch closure as compared to the control cells. Control untreated cells closed 52.1 ± 7.4% scratch in the first 12 h, whereas QA-treated cells closed 30.5 ± 9.0% scratch (*p* < 0.05). After 24 h, control cells closed 81.3 ± 10.2% of the scratch, whereas QA-treated cells barely showed any better closure than what it was at 12 h with a 41.0 ± 7.5% closure (*p* < 0.01) (Figure 2D). This indicates that QA is potent regulator of cellular migration, which could be an added asset to QA’s arsenal in bettering MPM prognosis.

#### 2.1.4. QA Successfully Traverses across Cell Membrane and Localizes around the Nucleus of MPM Cells

**Figure 3:**
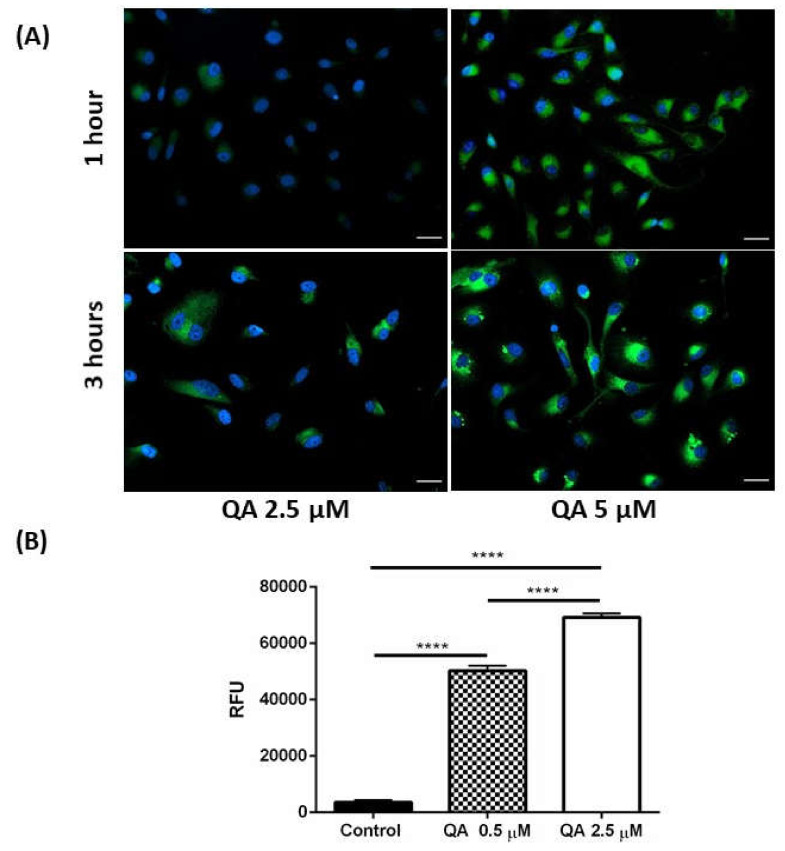
Evaluation of cellular internalization of QA by visual estimation and flow cytometry. (**A**) Cellular uptake study with QA (2.5- and 5-µM)-treated H28 cells. Nucleus is stained blue with DAPI. QA is indicated in green owing to its inherent fluorescence. It can be seen that there is a concentration and time-dependent uptake of QA in H28 cells. Increase in concentration and exposure time increases the internalization of QA, represented by the increase in fluorescence intensity. (**B**) Plot represents fluorescence intensity (F.I.) of QA as measured using flow cytometer. Briefly, MSTO-211H cells (500,000 cells/well) were exposed to QA (0.5- and-2.5 µM) for 3 h and F.I. was measured. A significant increase in F.I. is observed with increase in concentration. Data represents mean ± SD (*n* = 3), **** *p* < 0.0001, compared between treatments and control groups, as indicated, Scale bar 100 µm.

After evaluating the cytotoxic potential of QA, it is very essential to discover the intracellular localization of QA before deep diving to understand the molecular mechanisms against MPM proliferation. For this, cellular uptake studies were performed on H28 MPM cells by treating with QA (2.5- and 5-µM) at two time points (1- and 3-h). As QA is inherently fluorescent, it did not require a fluorescent tag for analysis of intracellular permeation. A concentration dependent intracellular uptake was observed as higher concentration QA (5 µM) was internalized more as compared to lower concentration (2.5 µM) QA at the same time interval, as represented in Figure 3A. Apart from this, 3-h incubation resulted in higher accumulation of QA inside the cells, revealing a time-dependent internalization. Cells’ nuclei were labeled with DAPI nuclear stain, while QA was observed to predominantly have a cytosolic localization.

Cellular uptake of fluorescent QA (0.5- and 2.5 µM) was also quantified by flow cytometry (FlowSight, Luminex Corp., Austin, TX, USA) following 3-h incubation; and a concentration dependent increase in fluorescence intensity was observed as seen in Figure 3B, indicating enhanced cellular internalization of QA. Higher concentration of QA resulted in a ~19 times increase in fluorescence intensity as compared to control, whereas, lower concentration resulted in ~14 times increase in fluorescence intensity as compared to control (Figure 3B). This signifies QA’s excellent capability to traverse through the cellular membrane, and internalize in the cells, without a need for a delivery system. It is well known that QA is a cationic drug [42] and has a very high pKa (9.4 and 10.7), which classifies it as a weak base. QA has also been reported to remain ionized at physiological pH [42]. Ionized species do not traverse the negatively charged cell membrane via passive diffusion and thus there has to be another potential mechanism of QA uptake in cells. One of the proposed mechanisms for uptake of cationic compounds in cells is via the proton pump vacuolar (V)-ATPase channel [43,44]. It has also been reported that mesothelioma cells express V-ATPase proton pumps [45]. Thus, excellent cellular internalization of this cationic charged molecule at physiological pH can be attributed to the uptake and retention of QA in cells via the proposed proton pump channel.

#### 2.1.5. QA Exhibits Excellent Efficacy in an In-Vitro 3D Spheroid Model of MPM Cells Mimicking Physiological Tumors

**Figure 4:**
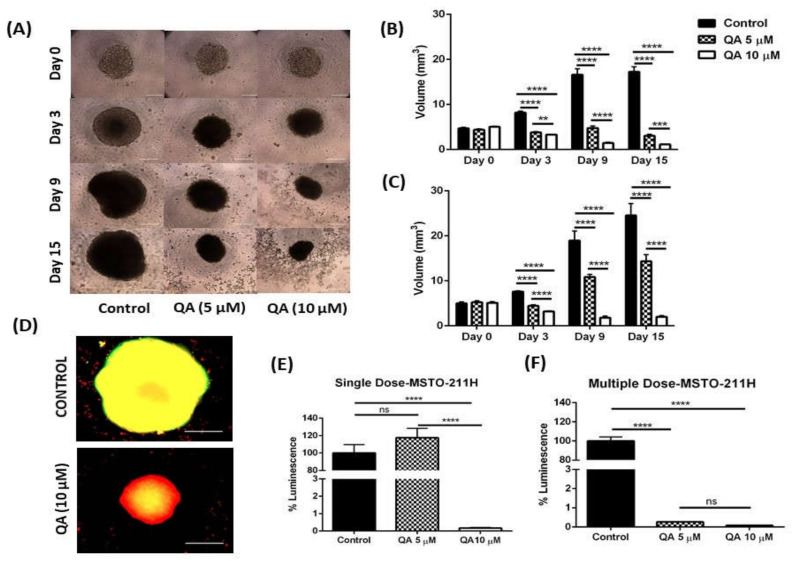
Three-dimensional-spheroid cell culture study for QA in MSTO-211H cell line. (**A**) Represent spheroids from multiple dose regimen for MSTO-211H cells, representing day 0, 3, 9, and 15. It can be clearly seen that increase in concentration of QA (5- and 10 µM) and exposure time, inhibition of spheroid growth is increased. MSTO-211H spheroids show a huge difference in the spheroid size over 15 days, with control spheroids reaching sizes as big as 25 mm^3^ in volume, whereas treated spheroids are as small as 1 mm^3^. (**B**) Plots represent spheroid volumes plotted for multiple dose and (**C**) single dose treatments for both cell lines. A significant difference can be seen in almost all treatment groups and control for all 3 days and both dosing regimens. (**D**) Representation of Live/Dead cells in the spheroid mass. Live cells are stained green and dead cells are stained red using a live/dead cell assay kit. It can be clearly seen that QA-treated spheroids have more dead cells than untreated spheroids, indicating excellent potency. (**E**,**F**) Plots represent cell viability of MSTO-211H spheroids after both single and multiple dosing regimen. Data represents mean ± SEM (*n* = 6) for volume and mean ± SD (*n* = 3) for viability analysis, ** *p* < 0.01, *** *p* < 0.001, **** *p* < 0.0001, compared between treatment and control groups, as indicated, Scale bar 1000 µm.

**Figure 5:**
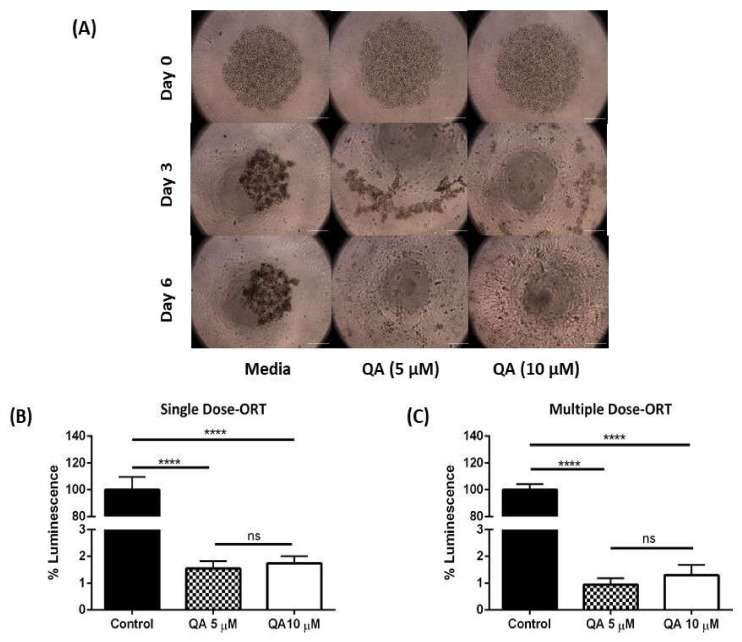
Three-dimensional-spheroid cell culture study for QA in primary patient-derived ORT cell line. (**A**) Represent spheroids from multiple dose regimen for ORT patient-derived cells, representing day 0, 3, and 6. It can be seen from the images that ORT spheroids are incapable of forming intact and sturdy spheroids and instead form loose clusters of cells. Hence visual estimation was terminated at the end of day 6. (**B**,**C**) Plots represent cell viability of ORT spheroids after both single and multiple dosing regimen. A significant difference between viability of cells between untreated and QA-treated spheroids can be clearly seen. Data represents mean ± SD (*n* = 3), **** *p* < 0.0001, compared between treatment and control groups, as indicated, Scale bar 1000 µm.

All the above-mentioned efficacy studies were performed in-vitro on a 2D monolayer of cells, which unfortunately does not accurately represent/mimic tumor growth within complex physiology of human body. Complete reliance on 2D in-vitro studies have resulted in several failed lead compounds, and QA’s efficacy also may not translate in preclinical studies. Keeping this in mind, three dimensional (3D) spheroids were tested for QA’s tumor regression potential, so as to simulate physiological tumor growth [46]. Multiple studies have been performed to confirm the utility of 3D spheroids in evaluating efficacy of treatment groups in physiologically relevant tumor models [47]. A 3D spheroid model not only provides close simulation to physiological conditions but also helps in high throughput screening of treatment doses, which can then be hand-picked for pre-clinical testing [48]. In this study, two MPM cell lines (1 immortalized cell line MSTO-211H and 1 primary cell line ORT) were tested for their ability to grow as solid tumors; and QA’s efficacy in attenuating tumor growth was studied. While MSTO-211H developed very well shaped and sturdy spheroid tissues (Figure 4A), ORT failed to form intact spheroids with loosely attached cellular aggregates in the culture wells (Figure 5A). QA treatment (5- and 10-µM) was added in two forms of dosing, a single-dose and a multiple-dose (dose replenishment every 72 h), as established in our earlier studies [28]. Media replacement was done every 72 h. Single dose treatment mimics the conditions routinely encountered in cytotoxicity studies, whereas multiple dose simulates chemotherapeutic regimens where patients are chronically administered multiple doses of the oncolytic agents. Figure 4A represents MSTO-211H spheroids on a multiple dosing regimen over a course of 15 days following treatment. Spheroid diameters were calculated using ImageJ open source software, followed by calculation of spheroid volumes. Spheroid volumes for each group were compared to control spheroids which did not receive QA therapy.

##### Visual Estimation—MSTO-211H Spheroids

Figure 4A represents MSTO-211H spheroids from a multiple dosing regimen of QA therapy over 15 days. It can be seen that the control group spheroids gradually grew in size over 15 days. On the other hand, growth for QA-treated spheroids was drastically inhibited, indicating excellent QA potency to attenuate tumor progression. All spheroid diameters were analyzed using ImageJ and volumes of spheroids were estimated from the analyzed diameters. Figure 4B represents plot for volumes of spheroids over 15 days of QA treatment (Multiple Dose). Prior to treatment, spheroid volumes for control spheroids and QA-treated spheroids for a multiple dosing regimen were as follows; Control: 4.7 ± 0.2 mm^3^, 5 µM QA: 4.4 ± 0.2 mm^3^_;_ and for 10 µM QA: 5.1 ± 0.2 mm^3^. No significant differences were observed between spheroid volumes at the beginning of this study (Figure 4B). As the study progressed to day 3, significant difference was observed in spheroid volumes with control spheroids being: 8.2 ± 0.3 mm^3^, whereas QA-treated spheroids were 3.8 ± 0.2 mm^3^ (5 µM QA; *p* < 0.0001 relative to control) 3.3 ± 0.2 mm^3^ (10 µM QA, *p* < 0.0001 relative to control). Similar significant results were obtained on day 9 for QA-treated MSTO-211H spheroids relative to the control. At the end of this study after 15 days, both 5µM QA (3.1 ± 0.3 mm^3^; *p* < 0.0001) and 10 µM QA (1.1 ± 0.2 mm^3^; *p* < 0.0001) treated spheroid volumes were significantly different than control spheroids (17.2 ± 1.1 mm^3^), indicating efficient and concentration dependent anti-cancer potency of QA therapy (Figure 4B). Similarly, when MSTO-211H spheroids were dosed only once at the beginning of 15 days (Single-dose study), following spheroid volumes and significant differences were observed (Figure 4C); Day 0: Control: 4.9 ± 0.3 mm^3^, 5 µM QA: 5.2 ± 0.3 mm^3^, 10 µM QA: 5.1 ± 0.2 mm^3^; Day 3: Control: 7.6 ± 0.2 mm^3^, 5 µM QA: 4.4 ± 0.3 mm^3^ (*p* < 0.0001), 10 µM QA: 3.2 ± 0.1 mm^3^ (*p* < 0.0001); Day 9:- Control: 19.0 ± 2.1 mm^3^, 5 µM QA: 10.9 ± 0.6 mm^3^ (*p* < 0.0001), 10 µM QA:1.8 ± 0.4 mm^3^ (*p* < 0.0001); Day 15:- Control: 24.6 ± 2.6 mm^3^, 5 µM QA: 14.4 ± 1.5 mm^3^ (*p* < 0.0001), 10 µM QA: 2.0 ± 0.3 mm^3^ (*p* < 0.0001) (Figure 4C). All *p* values are in comparison to respective no-treatment control groups. Figures S2 and S3 represent spheroids at all tested time points for single- and multiple-dosing, respectively, of MSTO-211H.

##### Live/Dead Cell Assay—MSTO-211H Spheroids

While microscopic imaging showed great promise, it still provided us with 2D quantification of a 3D structure (spheroid). Thus, visual representation of spheroids is insufficient to quantify the extent of treatment efficacy as there may be dead cells at spheroid core, which may not be accounted while measuring the size of spheroids. Thus, to visualize live and dead cells in the spheroid mass, a live/dead assay kit was used (Biotium, Fremont, CA, USA). This kit consists of two components, an esterase substrate calcein-AM, which measures metabolic activity and stains live cells green, and EthD-III, which measures the extent of necrosis by staining necrotic cells red. This fluorescent staining was visualized using Evos-FL fluorescence microscope (Life Technologies, Carlsbad, CA, USA) with green and red fluorescence protein filters. Figure 4D represents overlay fluorescent images of spheroids from multiple dose regimen for both cell lines following 15 days of treatment. It can be clearly seen that 10 µM QA-treated spheroids had higher intensity of red fluorescence as compared to the control (no treatment) spheroids in MSTO-211H, which have a yellow overlay. This yellow overlay is attributed to higher green fluorescence and lesser red fluorescence, indicating higher number of cells alive as compared to QA-treated spheroids. These results align with the visual representation of spheroids in the earlier section and further cement the excellent therapeutic potential of QA in MPM treatment.

##### Viability Analysis—MSTO-211H Spheroids

Along with microscopic imaging, a cell viability assay was performed using cell titer glo 3D^®^ assay kit (Promega Inc., Madison, WI, USA) to further establish cellular viability in in-vitro simulated tumor models. For MSTO-211H single dosed spheroids, a 117.5 ± 10.9% (5 µM QA; *ns to control*) and 0.2 ± 0.03% (10 µM QA, *p* < 0.0001 to control) viability relative to the control was observed (Figure 4E). For multiple dose spheroids, a viability of 0.3 ± 0.01% (5 uM QA, *p* < 0.0001 to control) and 0.1 ± 0.01% (10 µM QA, *p* < 0.0001 to control) relative to the control was observed (Figure 4F). It can be seen that effect of QA therapy is much more enhanced in viability analysis as compared to visual estimation. This can be attributed to the presence of dead cells in spheroid core [49,50], which are not accounted for in visual estimation. Hence, from live/dead and viability analysis, the potency of QA therapy can be judged. Viability analysis shows around a 500-fold difference between untreated spheroid growth and QA-treated spheroid growth, which is a very promising outcome for MPM therapy. The results obtained from both viability and live/dead analyses further cement the case for QA monotherapy to be a viable alternative to conventional chemotherapy.

##### Visual Estimation—ORT Spheroids

Figure 5A represents wells seeded with ORT cells for spheroid formation. Day 0 images indicate that ORT cells were loosely attached and did not form intact spheroids after overnight incubation. This was unlike MSTO-211H spheroids, which formed intact sturdy spheroids after overnight incubation. As experiment progressed for ORT spheroids following QA treatment, there was no improvement in formation of distinct core and boundary in spheroids, indicating that ORT cells were not suitable for 3D cell-based studies. However, impact of QA treatment could still be visually observed. As can be seen on day 6, control group spheroids were still loosely attached but QA-treated spheroids were completely dissipated. This complete dissipation can be attributed to QA therapy (Figure 5A). Visual estimation was halted after 6 days of treatment due to lack of any cellular structure in QA-treated wells. Due to absence of well-defined boundaries for spheroids, no measurements were performed in ORT cell spheroids; and we proceeded with cell viability analyses.

##### Viability Analysis—ORT Spheroids

Even though no intact spheroids were formed for ORT cells, viability of cells could still be tested, and results revealed that QA-treated single dosed spheroids had a viability of 1.5 ± 0.3% (5 µM QA, *p* < 0.0001 to control) and 1.7 ± 0.3% (10 µM QA, *p* < 0.0001 to control) relative to the control (100% viability; Figure 5B) and multiple dose spheroids had 0.9 ± 0.2% (5 µM QA, *p* < 0.0001 to control) and 1.3 ± 0.4% (10 µM QA, *p* < 0.0001 to control) viability as compared to control (Figure 5C).

A pre-clinical study involving cisplatin and pemetrexed in mesothelioma induced BALB/c HanHsd-Prkdc SCID mice revealed that a daily dose of cisplatin and pemetrexed had minimal effect on tumor growth inhibition and the growth aligned with no treatment control group of mice over 30 days [51]. On the other hand, a single dose of QA therapy helped significantly inhibit tumor growth inhibition in-vitro multiple folds as compared to untreated control. The overall results reveal the potency of QA in a 3D cellular model, which better mimics the physiological tumor formation and progression than 2D models. Though this model is highly established at the moment, it is still not close to replacing animal models due to either being over-simplistic or failing to replicate the interaction between cells and physiological system [52]. Instead, this experiment can be used as a bridge to gap in-vitro and future in-vivo studies to help in optimizing effective preclinical doses to be tested in animal models, thus limiting the number of animals and thereby reducing drug development costs. The results obtained in this extensive 3D spheroid study hint that QA therapy maybe the go-to-therapy with low doses and pre-established safety parameters. Further pre-clinical testing will be needed to accurately predict the therapeutic potential and dosing strategy for QA therapy in MPM.

### 2.2. Evaluation of Potential Mechanism of Action for QA Therapy in MPM Treatment

#### QA Successfully Demonstrates Anti-Angiogenic Traits in MPM Cells

**Figure 6:**
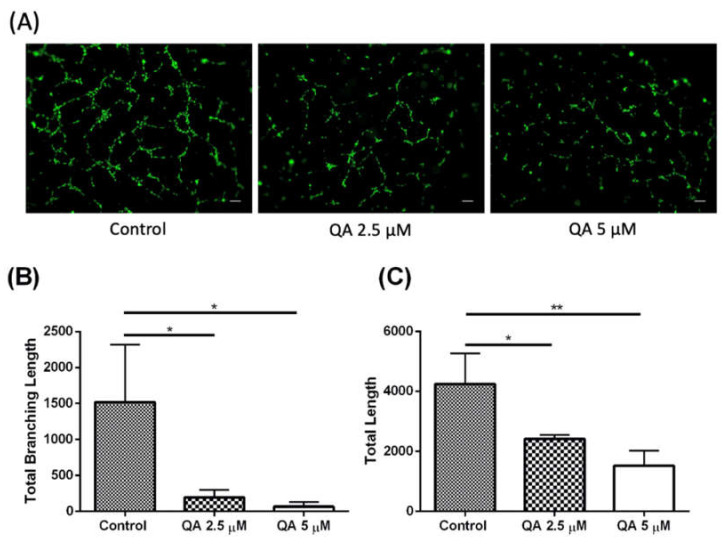
Evaluation of QA efficacy in inhibition of angiogenesis process in human umbilical vein endothelial cells (HUVEC)**.** (**A**) As compared to control, it can be seen that increasing concentration of QA inhibits tube formation in HUVEC. Highest concentration of QA barely permitted the formation of tubes, which is essential in nutrient provision in tumors. (**B**) Plot represents total branching length in HUVEC after treatment with QA at two concentrations. A significant difference in branching length can be seen as the concentration of QA is increased. (**C**) Plot represents total tube length in HUVEC after treatment with two concentrations of QA. A significant difference is seen in total formed tube length between QA-treated and control group of cells. Data represents mean ± SD (*n* = 3), * *p* < 0.05, ** *p* < 0.01, compared between treatment and control groups, as indicated, Scale Bar 200 µm.

The above discussed studies establish QA’s excellent phenotypic efficacy in MPM treatment. Once the efficacy was established, it was very important to understand QA’s underlying mechanisms of action for efficacy against MPM. We hypothesized that QA acted against MPM via multiple pathways, thus acting as a polypharmacological agent [53]. One of the pathways discovered was the effect on angiogenesis [54]. Angiogenesis process was first identified in 1970s to be exclusive to cancerous cells, hypothesizing it to be a mechanism of new blood vessels formation by cancerous cells for nutrient provision and sustainability [55]. It has been reported that angiogenesis, along with various cellular signaling pathways like fibroblast growth factor pathway, contribute toward rapid cell proliferation and division [56]. Hence, inhibition of angiogenesis would be detrimental for cancer cell survival. Angiogenesis has also been pivotal in highly vascularized tumors such as MPM, and it has been reported that MPM is characterized by multiple angiogenic markers and factors, like vascular endothelial growth factors (VEGF) and fibroblasts growth factors FGF-1 and FGF-2, that contribute toward MPM progression [57]. Multiple clinical reports reveal angiogenesis as potential target for MPM treatment with moderate to excellent success rates in attenuating MPM progression [58,59,60]. Keeping this and previous reports about QA’s effectiveness against tumors via affecting the angiogenesis pathway in mind [54,61], QA was tested for its potential use as an angiogenesis inhibitor in MPM. An in-vitro angiogenesis assay was performed using Cultrex^®^ in-vitro angiogenesis assay tube formation kit (R&D Systems, Minneapolis, MN, USA) in human umbilical vein endothelial cells (HUVEC) as model cells. As can be seen from Figure 6A, a concentration dependent inhibition in tube formation is evident on treatment with QA as compared to control HUVEC cells, which were freely and widely involved in formation of tubes for their sustainability and nutrient enrichment. Figure 6B,C represent the branching length and total length of the formed tubes, respectively, quantified using ImageJ software. In the presence of angiogenic factors, like VEGF or fibroblasts growth factors (FGF)-1 and FGF-2, endothelial cells can form capillary like structures (tubes) for nutritional supply and these tubes can be quantified in a way, first described in 1988 [62]. Quantification of tube length signifies the extent of angiogenesis and quantification of branching of these tubes can indicate the complexity of these tubing networks [63]. As can be seen in Figure 6B, branching length for QA-treated cells was 192 ± 109 units for 2.5 µM QA and 65.7 ± 65.7 units for 5 µM QA treatment, as compared to control cells with 1517.7 ± 799.4 units of tube branching. A similar trend was observed for total tube length with control cells exhibiting a total tube length of 4240 ± 1784 units, whereas QA treatment attenuated the total tube length with 2419 ± 223.3 (2.5 µM QA) units and 1522 ± 867 (5 µM QA) units of length (Figure 6C). Angiogenesis has been deemed very crucial for MPM survival and newer clinical therapies have aimed at targeting this particular pathway [64]. A category 1 FDA approved therapy involves a combination of cisplatin and pemetrexed, along with bevacizumab (VEGF monoclonal antibody; Avastin^®^), for patients which underwent no prior treatment for MPM, and this therapy specifically targeted angiogenesis pathway. An improvement in survival of patients was achieved with this newer therapy pushing the survival to 18.8 months compared to 16.6 month by chemotherapy [65]. This noteworthy elongation of survival indicates that inhibition of angiogenesis can be one of the added attributes of QA in inhibition of MPM growth.

### 2.3. QA Inhibits the Autophagy Process Thereby Potentially Starving Cells of Essential Nutrients and ATP

**Figure 7:**
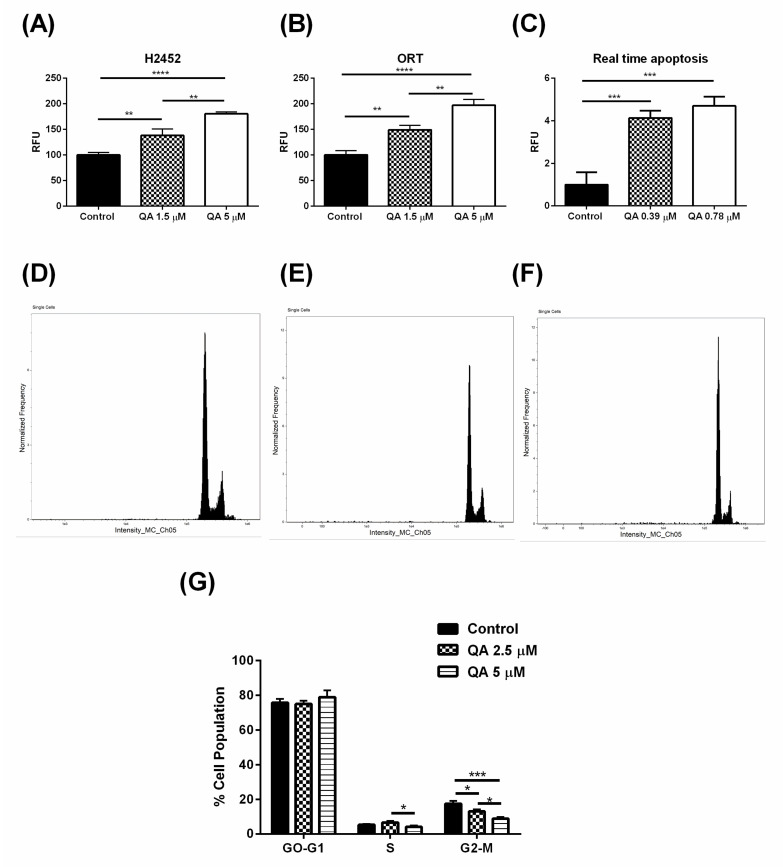
Representation of QA’s effect on autophagy and apoptosis pathways along with its effect on cell cycle in MPM cells. (**A**) Representation of autophagy inhibition performed by an autophagy assay kit in H2452 cells. It involves use of a probe that specifically binds to the surface LC3B protein present on an intermediate substrate in the autophagy process. Increase in fluorescence intensity represents higher degree of intermediate than the final product in autophagy process, indicating higher inhibition of autophagy H2452 cells. (**B**) Similar results were observed in patient-derived ORT cells where LC3B-2 expression levels were upregulated for QA-treated cells. (**C**) Plot represents in-vitro assay for determination of apoptosis in MM cells. Real time apoptosis study was performed for 3 h where an increase in levels of Annexin V were observed as QA concentration was increased (0.39- and 0.78 µM). (**D**–**F**) Plots represent various phases of cell cycle for control cells and QA-treated cells, as analyzed using FlowSight flow cytometer. (**G**) Plot represents population of cells in various phases of cell cycle, obtained by measuring DNA content by PI staining using flow cytometer. A significant difference can be seen in the population of cells in the G2-M phase as QA concentration is increased, indicating less population of cells are pushed towards mitosis and instead are arrested at the apoptotic stage. Data represents mean ± SD (*n* = 3), * *p* < 0.05, ** *p* < 0.01, *** *p* < 0.001, **** *p* < 0.0001, compared between treatment and control groups, as indicated.

QA is widely reported to affect (inhibit) the autophagy pathway in cells leading to deprivation of essential cellular nutrients in stressful conditions [28]. Autophagy is a process which involves the breaking down of macromolecules and cellular organelles into smaller monomer units by autophagic vesicles [30]. These monomer units are then recycled providing essential nutrients and energy to cells during stressful conditions [66]. This process is relevant in various conditions apart from cancers like atherosclerosis [67], endometriosis [68], dermatological conditions [69], neurodegenerative diseases [70], etc. The common link between all these conditions involves stressful cellular conditions, which forces cancer cells to recycle their own constituents in order to survive [30]. Similar adverse conditions are faced by MPM cells physiologically; and autophagy has been extensively reported to play a vital role in MPM survival [71]. Similar to known autophagy inhibitor chloroquine, QA has also been reported to act on the late phase of autophagy via inhibition of autophagosome-lysosome fusion, an essential step which leads to formation of aforementioned autophagic vesicles [28]. Inhibition of this fusion leads to accumulation of an essential autophagic marker LC3B-2 [72]. Autophagy inhibition thus can be studied via studying the expression levels of this specific autophagic marker (LC3B-2). In this study, an in-vitro CYTO-ID^®^ Autophagy Detection Kit (Enzo Life Sciences Inc., Farmingdale, NY, USA) was used to study the expression levels of LC3B-2. Contents of the kit were curated to fluorescently label LC3B-2 specifically and an increased signal output confirmed accumulation of LC3B-2 in cells. Effect of QA on autophagy was investigated in one primary (ORT) and one immortalized (H2452) cell line; and fluorescence signals received following QA treatment were compared to control MPM cells with no treatment. As seen in Figure 7A,B, in both H2452 and ORT cells, fluorescent signal increased significantly relative to control cells in a concentration dependent manner with QA treatment (5 µM > 1.5 µM QA). This signifies inhibition of autophagosome/lysosome fusion leading to inhibition of LC3B-2 degradation, hence its higher accumulation as compared to control cells. Relative to control, in H2452 cells, there was a 1.3-time increase (*p* < 0.01) in fluorescence intensity for 1.5 µM QA-treated cells and 1.8 times increase (*p* < 0.0001) in intensity for 5 µM QA-treated cells (Figure 7A). Similar results were observed for ORT cells with ~1.5 times (*p* < 0.01) increase in intensity for 1.5 µM QA-treated cells, and a ~2 time (*p* < 0.0001) increase for higher concentration of QA as compared to the control (Figure 7B). These findings strongly hint that a potential pathway for QA therapy can be inhibition of the autophagy process leading to starvation of cells of nutrients essential for survival. Another report suggested efficacy of a combinatorial therapy of pemetrexed and simvastatin for MPM treatment via inhibition of autophagy [73]. Several reports also link PI3k/mTOR inhibitors to be effective against MPM due to inhibition of autophagy process with or without a supplemental chemotherapy [74,75]. These reports suggest that inhibition of autophagy plays a vital role in attenuating MPM progression, making QA therapy even more relevant.

### 2.4. QA Therapy Leads to Induction of Apoptosis in MPM Cells

QA successfully inhibited autophagy, and to see its effect on apoptosis, an in-vitro assay was performed to check for the expression levels of Annexin V, a well-known apoptotic marker in cells. Annexin V levels were tested by using a RealTime-Glo™ Annexin V apoptosis and necrosis assay (Promega Inc., Madison, WI, USA). This assay was performed for 3 h and luminescence signals were recorded. An increase in luminescence signals indicated an increased expression of Annexin V. As seen from Figure 7C, as the concentration of QA increased (from 0.39 µM to 0.78 µM), levels of Annexin V significantly increased in a concentration dependent manner for both concentrations. After 3 h of treatment, Annexin V levels, were found to be 4.1 ± 0.3 times higher than control (0.39 µM QA, *p* < 0.001), whereas a 4.7 ± 0.4 times increase in Annexin V levels was observed with 0.78 µM QA compared to control (*p* < 0.001), indicating induction of apoptosis. However, there have been reports suggesting that usage of Annexin V as an apoptotic marker maybe misleading due to its false positive results and due to its inability to stain cells with severely damaged cellular membranes [76]. Thus, follow-up studies are required to confirm the effect of QA on apoptosis.

### 2.5. QA Arrests MPM Cells in G2-M Phase of Cell Cycle, Indicated by a Lower Cell Population

Having discovered the potential mechanism of action of QA in MPM (inhibition of the autophagy process and apoptotic induction), it is necessary to understand at what stage of the cell cycle process are the cells arresting and moving toward apoptosis, post-QA treatment. Cell cycle analysis was performed using FlowSight flow cytometer (Luminex Corp., Austin, TX, USA). DNA content was measured by propidium iodide (PI) staining, which can be used for estimating phases of cell cycle. Figure 7D–F reveal the cell cycle plots obtained after PI staining, as measured by FlowSight. A clear representation of three phases of cell cycle can be seen from the plots. Figure 7G indicates the population of cells in various stages of the cell cycle, i.e., G0-G1 phase, S phase, and G2-M phase. Post G2-M phase, the cells differentiate into daughter cells and undergo mitosis. Plot representing cell cycle reveals that population of QA-treated cells in G2-M phase (13.1 ± 1.2% for 2.5 µM (*p* < 0.05) and 8.8 ± 1.1% for 5 µM (*p* < 0.001)) is significantly less than control cells (17.5 ± 1.5%), representing lesser cell division after QA treatment. Lower cell population in G2-M phase may also be a result of increased apoptosis post-QA treatment (Figure 7C). Quinacrine has been previously reported to be working on subG0/apoptotic phase and G2-M phase [28,77]. Our study reveals a lesser population in G2-M phase for QA-treated cells with higher population of apoptotic cells compared to the control. A G2-M phase arrest has been widely reported to be an indication that cell division has been controlled [78,79]. Along with this, our study reveals a significantly higher population of apoptotic cells, which is an indication of excellent therapeutic potential of QA.

### 2.6. QA Affects Autophagic and Apoptotic Markers in MPM Cells

**Figure 8:**
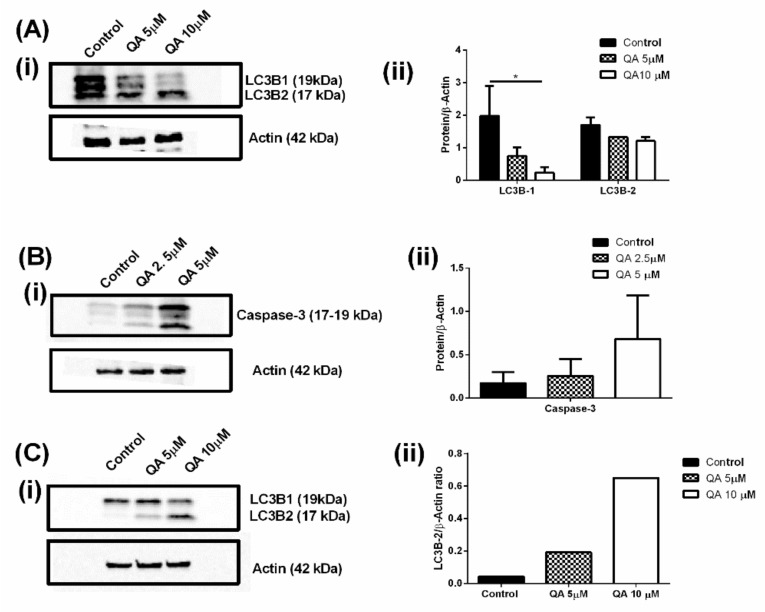
(**A**)-i Western blots representing the expression level of two subtypes (1 and 2) of LC3B protein post QA treatment (5- and 10 µM, 24 h) in MSTO-211H cells. It can be clearly seen that LC3B-1 is downregulated and LC3B-2 is upregulated in MSTO-211H cells, indicating successful inhibition of autophagy. (**A**)-ii Plot represents band intensity ratio for protein and B-actin for LC3B protein subtypes in MSTO-211H cells. A significant difference can be seen for LC3B-1 downregulation for QA treated cells as compared to control cells. (**B**)-i Western blot representing expression level of caspase-3, which is overexpressed in apoptotic events. It can be clearly seen that QA treatment (2.5- and 5 µM, 72 h) induces levels of caspase-3, indicating successful apoptosis. (**B**)-ii Plot represents band intensity ratio for protein and B-actin for caspase-3 in MSTO-211H cells. An upregulation of Caspase-3 marker can be seen as QA concentration is increased, indicating induction of apoptosis. (**C**)-i Blots represent LC3B expression levels in H2452 cells for control cells and QA treated cells. A clear induction in LC3B-2 levels are observed whereas LC3B-1 levels are seen to be downregulated. (**C**)-ii Plot represents band intensity ratio for protein and B-actin for LC3B-2 in MSTO-211H cells. Data represents mean ± SD (*n* = 3), * *p* < 0.05, compared between treatment and control groups, as indicated.

In-vitro autophagy assay was confirmed by checking the expression of LC3B protein in MSTO-211H and H2452 cells using western blotting. Autophagy inhibition has been deemed as a vital pathway for attenuating MPM progression, and measurement of LC3B levels has been one of the go-to techniques for analyzing therapeutic impact on autophagy process [30]. Two subtypes of LC3B were studied, LC3B-1 and LC3B-2. In an event of autophagy inhibition, levels of LC3B-1 are downregulated and LC3B-2 is upregulated. This is clearly visible in Figure 8(A-i) where LC3B-1 is downregulated and LC3B-2 is upregulated (24-h treatment) as QA concentration is increased. Band intensities were further quantified and plotted using ImageJ software and represented in Figure 8(A-ii). It can be seen that LC3B-1 levels in MSTO-211H are down significantly, as compared to control cells, represented as a ratio of LC3B-1/β-Actin, and this was found to be 2.0 ± 0.9 for control cells versus 0.7 ± 0.3 for 5 µM QA-treated cells and 0.2 ± 0.2 for 10 µM QA-treated cells. Induction of LC3B-2 can be attributed to QA’s successful disruption of autophagosome/lysosome fusion leading to inhibition of autophagy. Another apoptotic marker tested was caspase-3. As a proteolytic enzyme, caspase-3 is responsible for cleavage of DEVD peptide [80]. Caspase-3 has been reported to be extremely vital in both intrinsic and extrinsic cellular pathways and an apoptotic event leads to induction of caspase-3, which further disrupts multiple molecular pathways, including the disruption of cadherin-catenin complex [80]. The aforementioned induction of caspase-3 was evident after QA treatment (2.5- and 5 µM, 72 h), as seen in Figure 8(B-i). Quantification of caspase-3/β-actin ratio reveals that control cells had a ratio of 0.2 ± 0.1 versus 0.3 ± 0.2 for 2.5 µM QA-treated cells and 0.7 ± 0.5 for 5 µM QA-treated cells, indicating an induction in caspase-3 levels, thus indicating induction of apoptosis (Figure 8(B-ii)).

Effect of autophagy was also investigated in H2452 cells, which were used for in-vitro autophagy assay to evaluate whether the two discoveries were aligned. Figure 8(C-i) reveals blots for LC3B-1/2 expression in H2452 cells. It can be seen that LC3B-1 levels are declining with increase in QA concentration, along with simultaneous upregulation of LC3B-2 levels. LC3B-2 levels were quantified with control cells having an LC3B-2/β-actin ratio of 0.04 with 5 µM QA having a ratio of 0.2 and 10 µM QA having a ratio of 0.7, indicating higher accumulation of LC3B-2 and thus inhibition of autophagy (Figure 8(C-ii)).

## 3. Materials and Methods

### 3.1. Materials

Quinacrine dihydrochloride (QA) was procured from Sigma-Aldrich (St. Louis, MO, USA), cisplatin (CS) was obtained from Cayman Chemicals (Ann Arbor, MI, USA), crystal violet dye was obtained from Fisher Chemicals (Hampton, NH, USA), RPMI-1640 and Dulbecco’s Modified Eagle’s Medium (DMEM) cell growth medium along with sodium pyruvate, penicillin-streptomycin and trypsin were obtained from Corning (Corning, NY, USA), Fetal bovine serum (FBS) was obtained from Atlanta Biologicals (Flowery Branch, GA, USA). Vectashield hardset mounting medium with DAPI was procured from Vector Laboratories Inc. (Burlingame, CA, USA). Propidium iodide dye was obtained from Life Technologies (Carslbad, CA, USA), RNAse/DNAse was obtained from Thermo Fisher Scientific (Waltham, MA, USA). Pre-cast 4–20% TGX 15-well Gels and Midi PVDF transfer packs were obtained from Bio-Rad Laboratories (Hercules, CA, USA). Cell-titer blue viability assay kit and real-time apoptosis kit were obtained from Promega (Madison, WI, USA). Cultrex^®^ in-vitro angiogenesis assay tube formation kit was procured from R&D Systems (Minneapolis, MN, USA), and the CYTO-ID^®^ autophagy detection kit was obtained from Enzo Lifesciences (Farmingdale, NY, USA). Live/Dead viability assay kit was obtained from Biotium (Fremont, CA, USA). All other reagents were of analytical grade and were purchased from third-party vendors. Sources for all the antibodies are provided in respective sections.

### 3.2. Cell Lines Used and Culture Method

Two sets of MPM cell lines were used in this study, a set of immortalized cell lines, including MSTO-211H, H2452, H226, H28, and H2052 procured from American Type Culture Collection (ATCC, Manassas, VA, USA). To better represent MPM physiology, in-vitro testing was also done on primary patient-derived cell lines which included ROB, HAY, ORT, and YOU, procured from Dr. Raffit Hassan at the National Cancer Institute (Bethesda, MD, USA) under an executed materials transfer agreement and exempted IRB protocol 0818-034 from St. John’s University [81,82]. Human embryonic kidney cell line (HEK-293) was obtained from ATCC (Manassas, VA, USA). All cancer cell lines were cultured using RPMI-1640 cell growth medium supplemented with 10% Fetal Bovine Serum, 1% sodium pyruvate, and 1% penicillin-streptomycin, while HEK-293 were cultured in Dulbecco’s Modified Eagle’s Medium (DMEM) supplemented with 10% FBS, 1% sodium pyruvate, and 1% penicillin/streptomycin. Cell lines were grown at 37 °C/5% CO_2_ in tissue culture (TC) treated T75 cell culture flasks (Eppendorf, Hauppauge, NY, USA) until 80% confluency was reached. Confluent cells were trypsinized and used for in-vitro experiments. Immortalized cell lines were used until passage 20–25 and primary patient-derived cell lines were cultured up to 10 passages. QA stock for all treatments was freshly prepared prior to all experiments by dissolving the desired amount of QA in RPMI-1640 (DMEM for HEK-293) cell growth medium supplemented with 10% Fetal Bovine Serum, 1% sodium pyruvate, and 1% penicillin-streptomycin.

### 3.3. In-Vitro Phenotypic Efficacy of QA in Mesothelioma Cell Lines

#### 3.3.1. Cell Viability Studies

Cytotoxicity studies were performed by testing various concentrations of quinacrine (QA) and cisplatin (CS) on mesothelioma cell lines. Extensive cell viability studies were performed on five immortalized cell lines, four primary patient-derived cell lines, and 1 normal cell line over a range of concentrations. IC_50_ values for QA on each cancer cell line was determined using the non-linear fitting module available in GraphPad Prism ver. 6.0 (GraphPad, Inc., San Diego, CA, USA). Briefly, cell viability data in triplicates was transformed to log values and normalized to obtain IC_50_ values for QA on each cell line. Briefly, the cells were grown up to 80–85% confluency following the protocol listed above, and were plated in TC-treated 96-well plates (Eppendorf, Hauppauge, NY, USA) at a density of 2500 cells/well (6750 cells/cm^2^) and were incubated at 37 °C/5% CO_2_ overnight. The following day, cells were treated with varying concentrations of QA and CS; and were further subjected to a 72-h incubation in same conditions. After 72 h, cell viability was evaluated using cell-titer blue assay kit (Promega Inc., Madison, WI, USA) as per manufacturer’s protocol. Briefly, 20 µL of cell-titer assay reagent was added to treatments after the incubation period and the plates were incubated for another 2 h at 37 °C/5% CO_2_ followed by measurement of fluorescence intensity (540ex/590em) on Spark 10M plate reader (Tecan, Männedorf, Switzerland). Cell viabilities were calculated relative to no treatment control. All cell viability assays were performed in triplicates with *n* = 6 each time.

#### 3.3.2. Evaluation of Colony formation: Clonogenic Assay

In this assay, efficacy of QA to successfully inhibit MPM cell colony formation was evaluated. Briefly, H2452 cells were seeded in 6-well TC-treated plates (500 cells/well); and were allowed to attach overnight. H2452 cells were selected based on previous reports demonstrating successful colony formation [39]. The following day, growth media was replaced with multiple concentrations of QA (1.5- and 5-µM), and a control (growth media only) group was maintained. After 48 h, treatments were removed, and all wells were supplied with fresh growth media. Cells were further incubated for 7 days, with media replenishment every 48 h. After 7 days, media was aspirated, and cells were fixed (4% paraformaldehyde), followed by staining with crystal violet dye (0.01%) for colony visualization. Images were taken using a digital camera and were processed with a colony counter software, Open CFU, for counting individual colonies [83].

#### 3.3.3. Evaluation of Cellular Migration: Scratch Assay

Scratch assay is instrumental in evaluating cellular migration by studying migration of confluent cells across an artificially created scratch on a confluent monolayer of cells. In this assay, H226 cells were seeded in 24-well TC-treated plates (100,000 cells/well) and were allowed to form a confluent layer. H226 cells were selected based on previous reports demonstrating their utility in scratch assay [84]. Once confluent, a scratch was drawn across the wells with a *p200* sterile pipette tip, as reported earlier [85]. All wells were imaged for pre-treatment scratch conditions, followed by images at 12- and 24-h post-QA treatment (1.5 µM). Scratch area was calculated using ImageJ software ver. 2.0 (National Institutes of Health, Bethesda, MD, USA) and analyzed for all treatment groups.

#### 3.3.4. Evaluation of Cellular Internalization of QA in MPM Cells

Cellular internalization studies were performed to visualize the internalization of QA in H28 cells. This study demonstrates QA’s ability to traverse across the cell membrane primarily via passive diffusion. H28 cells were selected for this assay based on their elongated morphology and high visual appeal. Briefly, H28 cells were trypsinized and seeded in TC-treated 8-chambered cover glass (Eppendorf, Hauppauge, NY, USA) (10,000 cells/chamber). Cells were incubated and allowed to attach and grow overnight. The following day, growth medium was aspirated, and cells were treated with QA (2.5- and 5-µM) followed by incubation at 37 °C/5% CO₂ for (i) 1-, and (ii) 3-h. At each time interval, cells were fixed using 4% paraformaldehyde (PFA) for 10 min. PFA was then aspirated and the chambers were washed with ice-cold PBS 3 times. Next, chamber from the cover glass was carefully removed. Microscopic slide was prepared by placing vectashield hardset mounting medium with DAPI (Vector Laboratories, Burlingame, CA, USA), followed by carefully inverting the cover glass over slide, so as to avoid bubble formation. The slide was then allowed to sit overnight at 4 °C to harden the mounting medium. Cells were then imaged using an EVOS-FL (Thermo Scientific, Waltham, MA, USA) fluorescence cell imaging system using a 20× magnification lens.

Quantitative analysis of fluorescence intensity after cellular internalization of QA was also performed using Amnis^®^ FlowSight imaging flow cytometer (Luminex Corp, Austin, TX, USA). Briefly, cells were treated with QA (0.5- and 2.5-µM) for 3 h followed by three washing cycles using ice-cold PBS, cellular harvesting and fixing by 4% paraformaldehyde for 10 min. The fluorescence intensity of internalized QA was measured (50,000 events/sample) and was compared to untreated cell control to account for cellular auto-fluorescence. Lower QA concentrations were selected to account for equipment sensitivity.

#### 3.3.5. QA Efficacy in In-Vitro 3D-Spheroids

Three-dimensional spheroid cell culture studies were performed based on previous reports [28]. Briefly, MSTO-211H and ORT cells were trypsinized on reaching ≈80% confluency; and were plated in ultra-low attachment 96-well U-shaped plates (2000 cells/well) (Corning, NY, USA) followed by overnight incubation. The following day, formed spheroids were treated with multiple concentrations of QA (5- and 10-µM). A 24-h seeding time was chosen to mimic a prophylactic version of spheroids where treatment is initiated prior to formation of intact spheroids as opposed to a therapeutic model which involves initiation of treatment after formation of intact spheroids [86]. A single dose (single dosing on day 1) and a multiple dose (dosing every 72 h) regimens were followed [46]. Treatments and media replenishment were done by replacing half of the well contents. Images were captured every 72 h using an inverted color microscope (Laxco, Mill Creek, WA, USA) for 15 days. At the end of treatment period, spheroids were visually analyzed using ImageJ open source software to measure spheroid diameter. Subsequently, spheroid volume and area were calculated using the estimated diameter. Further, cell viability was measured using cell titer-glo^®^ 3D (Promega Inc., Madison, WI, USA), as reported earlier [87]. Briefly, 100 µL out of 200 µL of treatments and nutrient media present in spheroid wells was carefully removed and 100 µL of cell titer-glo^®^ 3D reagent was added. The plate was shaken vigorously for 5 min, followed by 30-min incubation at room temperature in dark. Luminescence measurement was carried out after incubatory period using Spark 10M plate reader and % cell viability was calculated for all treatment groups relative to the viability of control spheroids.

#### 3.3.6. Live/Dead Cellular Assay

At the end of treatment period (day 15) for previously grown MSTO-211H spheroids, live/dead assay was performed using a viability/cytotoxicity assay kit for live/dead cells (Biotium, Fremont, CA, USA) as per manufacturer’s protocol. Green stain (calcein AM) was used to stain alive cells, whereas red stain (ethidium homodimer III (EthD-III)) was used to stain the dead cells. Briefly, treatment groups and nutrient media was aspirated from the wells carefully, followed by addition of 200 µL of live/dead reagent, a cocktail of calcein AM and EthD-III, prepared as per manufacturer’s protocol. The plate was incubated in dark for 30 min. Further, fluorescent spheroids were imaged using an EVOS-FL fluorescence microscope (Thermo Scientific, Waltham, MA, USA) using GFP (green fluorescence protein) and RFP (red fluorescence protein) filters, respectively, at 20× magnification. Images from both red filter and green filter were overlaid to evaluate the dominance of dead cells versus live cells.

### 3.4. Mechanistic Evaluation of QA’s Anti-Mesothelioma Efficacy

#### 3.4.1. Evaluation of QA as an Angiogenesis Inhibitor

Angiogenesis is a key cellular mechanism which involves formation of new blood vessels from existing vasculature so as to fulfill the nutrient requirements of rapidly dividing tumorigenic cells [88]. It has been reported that patients with mesothelioma have higher expression of proangiogenic factors, like vascular endothelial growth factors (VEGF) and fibroblasts growth factors (FGF-1 and FGF-2), indicating its pivotal role in MPM [29]. QA is previously reported to be capable of attenuating angiogenesis in tumors [54], which can be a viable pathway for inhibiting MPM progression. In this assay, Human Umbilical Vein Endothelial Cells (HUVEC, Lonza, Basel, Switzerland) were seeded into a T25 flask and incubated overnight prior to performing angiogenesis assay using Cultrex^®^ Angiogenesis Assay Kit (R&D Systems, Minneapolis, MN, USA), as reported earlier [89]. The following day, 50 μL of Cultrex^®^ Reduced Growth Factor (RGF) Basement Membrane Extract (BME) was added to all test wells of a TC-treated 96-well plate and incubated (37 °C) for 60 min to allow the BME to gel. Simultaneously, HUVEC cells were stained with 2 μM calcein AM for fluorescence imaging for 30 min. After 30 min, these cells were trypsinized and seeded in BME-coated wells (1.3 × 10^4^ cells/well) along with multiple concentrations of QA (2.5- and 5-µM). The plates were incubated for 6 h at 37 °C/5% CO_2_ followed by imaging using EVOS-FL (Thermo Scientific, Waltham, MA, USA) fluorescence microscope. Branching length and total length of tube formation was evaluated and quantified using ImageJ software with the angiogenesis analyzer plug-in.

#### 3.4.2. Effect of QA on Cellular Autophagy

In this assay, CYTO-ID^®^ Autophagy Detection Kit (Enzo Life Sciences, Farmingdale, NY, USA) was used to determine the effect of QA on autophagy in MPM cells. Briefly, H2452 and ORT cells were seeded in 96-well plates (25,000 cells/well) and were incubated with serum-free media to induce starvation linked autophagy process for 24 h. After 24 h, starved cells were treated with QA (1.5- and 5-µM) for a further 18 h, as per manufacturer’s protocol. Treatments were then replaced with 1X assay buffer (100 µL) followed by addition of dual color detection reagent and incubated for 30 min in dark at 37 °C (100 µL of CYTO-ID^®^ Green Detection Reagent + Hoechst 33,342 nuclear stain growth medium without phenol red indicator supplemented with 5% FBS). Later, excess dye was removed by washing with 1X assay buffer and 100 µL of 1X assay buffer was added to each well. CYTO-ID^®^ Green Detection Reagent was read with a FITC filter (480 nm/530 nm ex/em), and the Hoechst 33,342 Nuclear Stain was read with a DAPI filter set (340 nm/480 nm ex/em) using Spark 10M plate reader.

#### 3.4.3. Cell Cycle and Apoptosis Analysis by Flow Cytometry

Cell cycle and apoptosis analysis was done using propidium iodide staining on Amnis^®^ FlowSight imaging flow cytometer (Luminex Corp, Austin, TX, USA). Briefly, MSTO-211H cells were seeded in TC-treated 6-well plates (500,000 cells/well, *n* = 3) and were incubated overnight for attachment. The following day, cells were treated with QA (2.5- and 5-µM) for 24 h. After 24 h, treatments were removed, and cells were washed thoroughly with 1X PBS, followed by trypsinization for cell harvesting. Cell pellets were collected and fixed with 70% ice-cold ethanol for 1 h. Following fixing, cells were washed with 1X PBS and incubated with RNA-ase (100 µg/mL) and propidium Iodide (10 µg/mL) for 30 min in dark. Cells were then analyzed (50,000 counts/sample) using Amnis^®^ Flowsight^®^ (Luminex Corp., Austin, TX, USA). Data were analyzed using IDEAS^®^ software ver. 6.2 (Luminex Corp., Austin, TX, USA).

#### 3.4.4. RealTime-Glo™ Annexin V Apoptosis and Necrosis Assay

The RealTime-Glo™ Annexin V Apoptosis and Necrosis Assay (Promega, Madison, WI, USA) is an assay that can measure apoptotic events based on Annexin V binding with phosphatidylserine, an extracellular apoptotic marker, in real-time. In this assay, MSTO-211H cells were plated at a density of 10,000 cells/well in a 96-well plate and were allowed to attach overnight, as reported earlier [86]. The following day, cells were treated with 4× concentration of QA (final concentration of 0.39- and 0.78-µM), followed by addition of 100 μL of 2× assay buffer. Treated plates were then incubated at 37 °C/5% CO_2_. Luminescence was measured at 0- and 3-h using Spark 10M microplate reader (Tecan, Männedorf, Switzerland).

#### 3.4.5. Western Blotting for Assessing QA’s Impact on MPM Molecular Markers

A previously established protocol for western blotting was used in this study [90]. Briefly, MSTO-211H cells were plated 1 × 10^6^ cells per petri dish (100 MPM diameter) and treated with QA (2.5-, 5-, and 10-µM) for 24 h at 37 °C/5% CO_2_. After treatment, the cells were collected and lysed with 1% Triton^®^ X-100 (Fisher Scientific, Waltham, MA, USA) and 1% Halt™ Protease and Phosphatase Inhibitor Cocktail (Thermo Fisher Scientific, Waltham, MA, USA) in PBS and sonicated for 1 h at 4 °C. Samples were centrifuged for 15 min at 4 °C at 15,000 rpm, and lysates were collected. Cell lysate protein was quantified by the DC™ Protein Assay Kit (Bio-Rad, Hercules, CA, USA). The samples were mixed with 2× Laemmli buffer (Bio-Rad) and 2- mercaptoethanol and denatured at 110 °C for 10 min. For Western blot analysis, protein was loaded at 10 µg and separated on 4–20% Mini-PROTEAN^®^ TGX™ Precast Protein Gels (Bio-Rad, Hercules, CA, USA) (135 mV for 60 min) and transferred to Trans-Blot^®^ Turbo™ Midi PVDF membranes (Bio-Rad, Hercules, CA, USA) using a Bio-Rad Trans-Blot^®^ Turbo™ transfer system. Membranes were blocked with 5% bovine serum albumin in PBS and spiked with LC3B (#3868S), β-actin (#8457S) (Cell Signaling Technology, Danvers, MA, USA); and caspase-3 (#700182) (Thermo Fisher Scientific, Waltham, MA, USA) primary monoclonal antibodies overnight at 4 °C. All primary antibodies were used at 1:1000 dilutions. Membranes were then incubated with the corresponding secondary HRP-conjugated antibodies; goat anti-rabbit (#32260) and goat anti-mouse (#31430) poly-HRP (Thermo Fisher Scientific, Waltham, MA, USA) (1:10,000 dilution) for 1 h at room temperature, and subjected to WesternBright chemiluminescence (Gel Company, San Francisco, CA, USA). Protein bands on the membranes were visualized using the Chemi mode by the Omega Lum™ G Imaging System (Gel Company, San Francisco, CA, USA). Densitometry for all protein bands was performed using ImageJ open source software ver. 2.0.

### 3.5. Statistical Data Analysis

All data presented here are mean ± SD or SEM (*n* = 3 to 6). Cytotoxicity studies represent average of 3 independent trials (*n* = 6 for each trial). To compare more than two groups, one-way ANOVA followed by Tukey’s post hoc multiple comparison test was used. *p* value < 0.05 was considered statistically significant (as mentioned at relevant places throughout the manuscript). All statistical calculations were performed on GraphPad Prism Version 6.0.

## 4. Conclusions

This study establishes the overall utility of an antimalarial drug, QA, in treatment of MPM by identifying multiple interdependent disease modifying pathways. The primary pathway of MPM inhibition is shown to be apoptotic induction and autophagic inhibition. This was extensively tested via multiple studies and a successful correlation was established between QA, autophagy and apoptosis. A secondary mechanism of QA activity is believed to be inhibition of angiogenesis, which will cut-off the supply of nutrients to the cancerous cells, leading to necrosis. All these mechanisms contribute synergistically resulting in excellent efficacy of QA against MPM, not only in the 2D cell culture but also in the 3D tumor spheroid model. Extensive in-vitro experiments performed in this study lay solid groundwork for QA to be a standalone therapy for MPM treatment. However, it is important to correlate findings from in-vitro studies to pre-clinical studies and one of the major obstacles is prediction of dose for animal models. Previous reports suggest that QA exhibits an IC_50_ value of around 1–2 µM which was successfully correlated to be equivalent to efficacious oral doses around 100–200 mg/kg in mice models [91,92]. However, there have been some oral bioavailability issues with QA administration, including high volume of distribution and accumulation in liver [93]. Thus, further studies can build on the current work and may involve formulating a drug delivery system to better this effect of QA and evade the oral bioavailability issues, along with extensive preclinical efficacy studies.

## Supplementary Materials

The following are available online at https://www.mdpi.com/1422-0067/21/17/6306/s1, Figure S1: Quinacrine (QA)’s Cytotoxicty in Normal Human Embryonic Kidney (HEK-293) Cells following incubation for 24- and 48-h. Data represents mean ± SD (*n* = 6), Figure S2: MSTO-211H spheroids on a single dose regimen, Figure S3: MSTO-211H spheroids on a multiple dose regimen.
