# Supplementary material for: Repurposing Quinacrine for Treatment of Malignant Mesothelioma: In-Vitro Therapeutic and Mechanistic Evaluation
Source: Int J Mol Sci. 2020 Aug 31;21(17):6306. doi: 10.3390/ijms21176306 (PMC7503636; doi:10.3390/ijms21176306)
Supplement: Supplementary file 1 [file ijms-21-06306-s001.pdf]

SUPPLEMENTARY MATERIAL

Repurposing Quinacrine for Treatment of Malignant Mesothelioma: In-vitro Therapeutic and Mechanistic Evaluation

Nishant S. Kulkarni, Bhuvaneshwar Vaidya, Vineela Parvathaneni, Debarati Bhanja, and Vivek Gupta

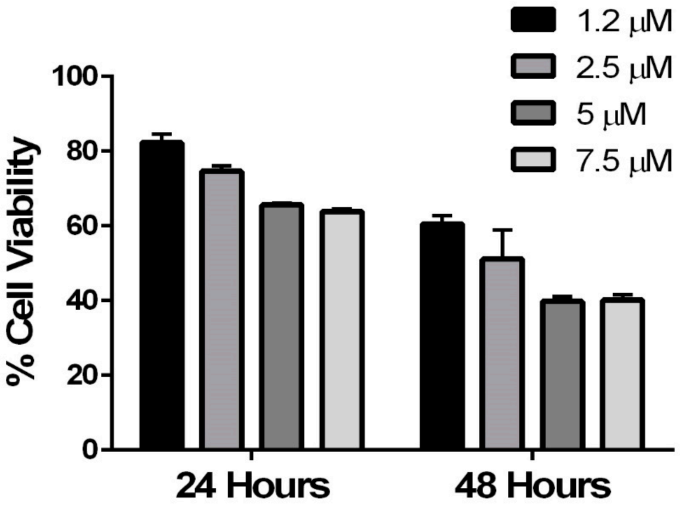

Fig. S1: Quinacrine (QA)'s cytotoxicity in normal human embryonic kidney (HEK-293) cells following incubation for 24- and 48-h. Data represent mean±SD (n=6).

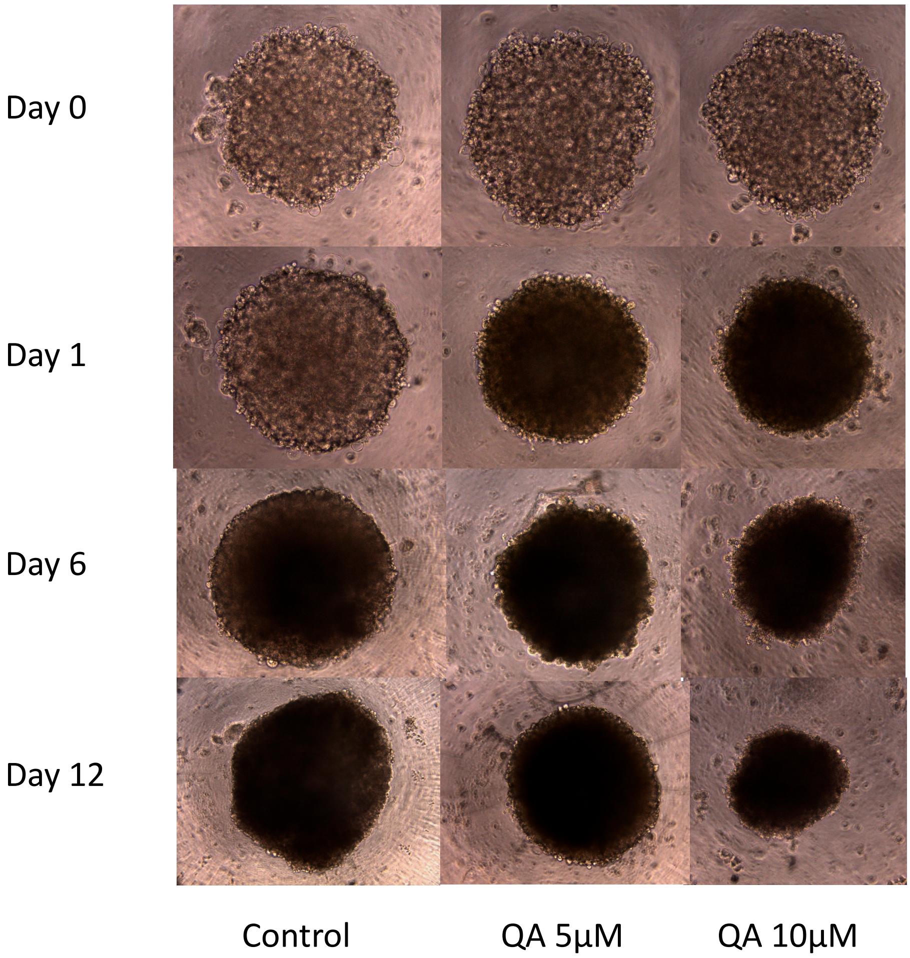

Fig. S2: MSTO-211H spheroids on a single dosing regimen.

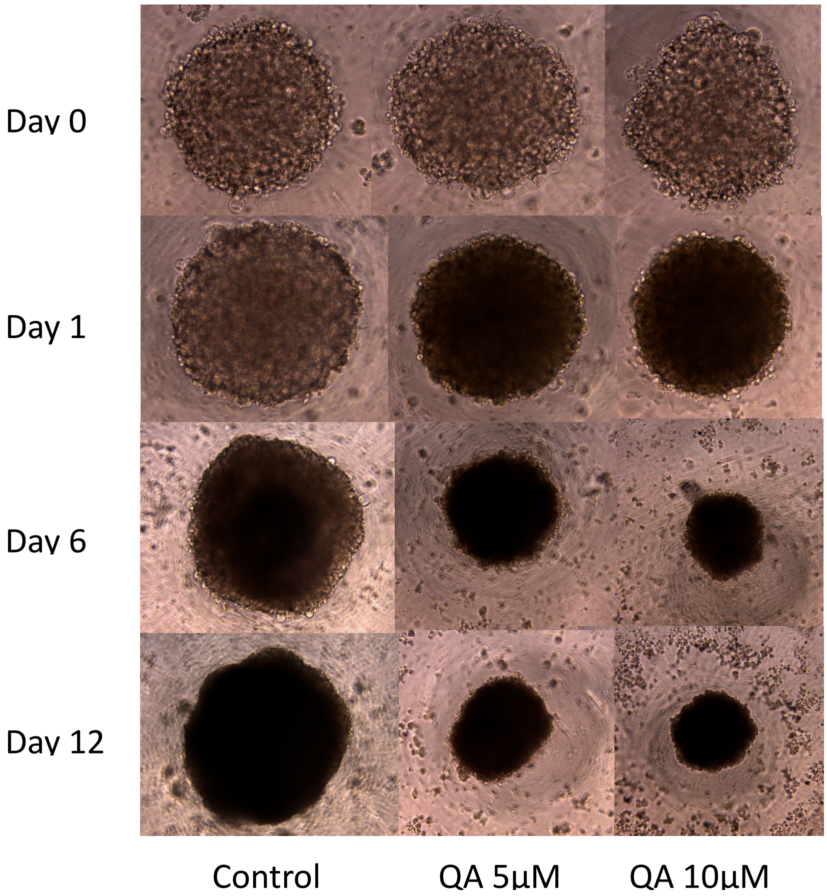

Fig. S3: MSTO-211H spheroids on a multiple dosing regimen.
